# Dr. Jagdish Ramnath Jaju: A Legend

**DOI:** 10.1055/s-0045-1812082

**Published:** 2025-10-31

**Authors:** Hemen Jaju

**Affiliations:** 1 Jaju Plastic Surgery Center, Ahmedabad, Gujarat, India

When I was asked to write about my father Dr. Jagdish Ramnath Jaju, I was perplexed. I had to view him through a kaleidoscope of relations that I had with him; he was first a father, then my mentor and guide; later, I joined him as a junior colleague and then was his helping hand in later life ( Fig. 1 ).

**Fig. 1:**
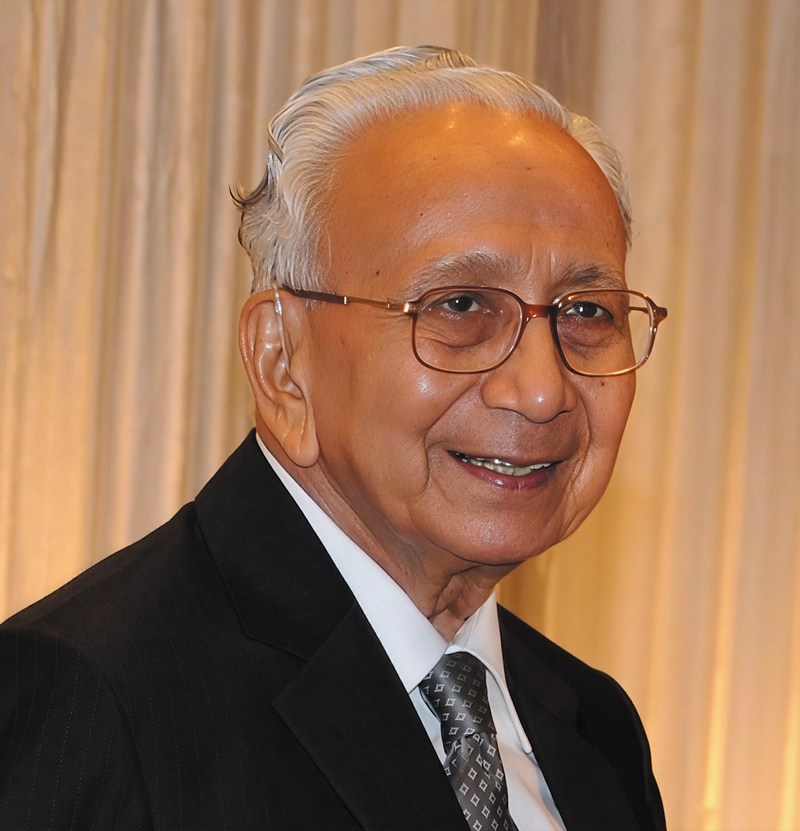
Dr. Jagdish Ramnath Jaju (1931–2023).

Plastic surgery in India, and particularly in the state of Gujarat, where Dr. J. R. Jaju is a pioneer, owes much to his vision, dedication, and excellence in the field. His commitment to patient welfare, innovation in surgical techniques, and service to the underprivileged, in rural and remote areas, has made him an iconic figure.

He was born on December 20, 1931, in a family of farmers, in Erandol, Jalgaon District, Maharashtra State, where he had early school education. He then moved to Fergusson College, Pune, for higher education, and in 1950 for MBBS at SSG Medical College, Baroda, Gujarat. He did his early general surgical training at SSG Hospital, Baroda and then went to the United Kingdom for pursuing and developing surgical skills further.

He received his FRCS (Eng) in 1959 ( Fig. 2 ). After his fellowship, he trained at the Birmingham Accident Hospital, United Kingdom, and later with Mr. Deitch at Halifax, Yorkshire, United Kingdom. Here he met and assisted Mr. Mortimer Shaw, an excellent craftsman doing reconstructive plastic surgery procedures. Inspired, he took up a registrar's post at St. James's Hospital, Leeds, United Kingdom, under Mr. Michael Oldfield and Mr. Mortimer Shaw; both had worked extensively with Sir Harold Gilles and Prof. Kilner at Rooksdown House, Basingstoke, during the war. After 3 years of training at St. James's Hospital, Dr. Jaju went for further training in hand surgery to Mr. Raoul Tubiana in France and for cleft lip and palate surgery training to Mr. Tord Skoog in Sweden.

**Fig. 2:**
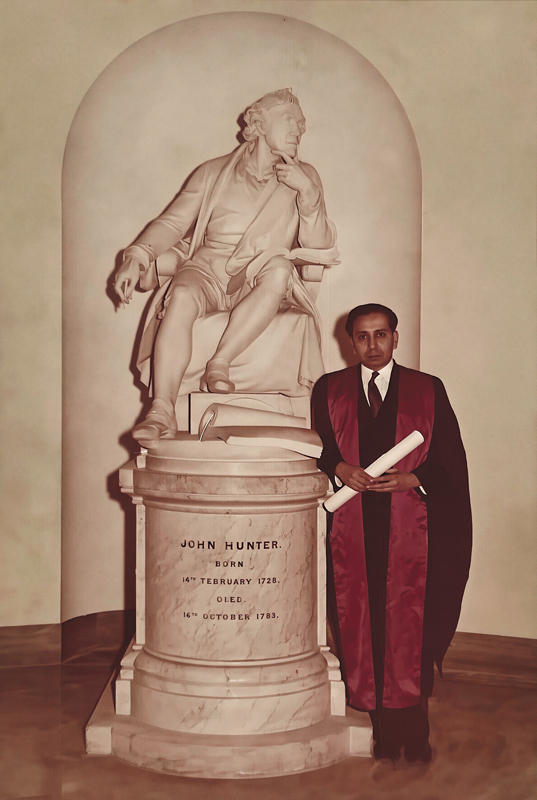
Dr. J.R. Jaju after receiving his FRCS (England) in 1959.

He declined a senior registrar's post at Leeds, to come back to India, and develop this specialty in our country, in an era when plastic surgery was at a nascent stage.

While he was in Leeds in 1963, he was offered a post of “Pool Officer” by the Council of Scientific and Industrial Research, both at Nagpur and Ahmedabad. He made the bold decision to base his practice in Ahmedabad, Gujarat at a time when there were no other plastic surgeons in Gujarat and even in the adjoining states of Rajasthan and Madhya Pradesh. This signified a commitment to bring plastic surgical care to underserved populations. In September1963, he accepted this post and joined B.J. Medical College and Civil Hospital, at Ahmedabad, the largest government teaching hospital in the country at that time. He was subsequently appointed as an Associate Professor. Dr. Jaju then established the first plastic surgery department in Gujarat state at Civil Hospital Ahmedabad in 1964. Late Dr. Anant D. Puranik joined him later as an assistant.

Late Prof. Goleria, from Mumbai, often recalled that during the early years in mid-1960's Dr. Jaju travelled to KEM Hospital, Bombay, for regular monthly plastic surgery meetings. Here, he developed a good rapport with the founder members of the Association of Plastic Surgeons of India (APSI) and became a member of APSI.

His pioneering spirit and enthusiasm to spread the reach of plastic surgery was evident in his eagerness and dedication for the following five years, seen in mass educating the doctors and common man in all the districts/subdistricts/taluka places in urban and rural Gujarat. This was done through the IMA College of General Practitioners, by way of audiovisual talks arranged every Sunday of the year, with very limited resources. Under his leadership, the field of plastic surgery in Gujarat saw a rapid rise, and the number of patients seeking the whole spectrum of plastic surgery reconstruction, increased rapidly; with a waiting list for planned surgery extending for many months. Under the Colombo plan in 1969–70, the department of plastic surgery got a centralized air-conditioned unit for patients, the first in the country at the time. At Civil Hospital Dr. Jaju met his MBBS and FRCS colleague, Dr. Dinubhai Patel, an orthopaedic surgeon, and together they worked as a team from 1965, in educating the doctors in Gujarat and emphasizing the complimentary role of these two specialties in treating patients. This teamwork lasted until the untimely death of Dr. Patel in 2007.

Dr. Jaju was very strict about ethics, sincerity, and discipline in work, and very compassionate with the patients. Late Prof. Udayan Vyas, who was his registrar in 1968–70, recalled that both his teachers, Dr. Jaju and Dr. Puranik, were dedicated surgeons, and everyone kept so busy caring for patients in the plastic surgery department, that often during their training period, they would not see the Sun above the horizon for days together ( Fig. 3 ).

**Fig. 3:**
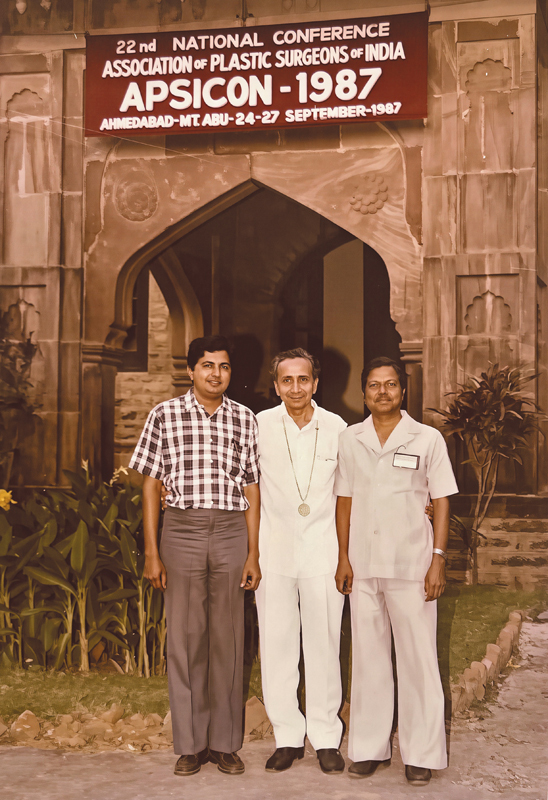
Dr. Udayan Vyas, Dr. J.R. Jaju, and Dr. Puranik at APSICON 1987 (L to R).

After working as the departmental head for 6 years at Civil Hospital, Ahmedabad, Dr. Jaju chose to move to private practice and continued his services at the Civil Hospital department, as an Hon. Professor till 1974–75.

During this time in 1974–75, the surgical unit commenced at Gujarat Cancer and Research Institute (GCRI), Ahmedabad. On being invited by Dr. T.B. Patel, then the director at GCRI, Dr. Jaju established the plastic surgery department at GCRI.

At GCRI, he regularly invited Mr. Bruce N. Bailey from Stoke Mandeville Hospital, United Kingdom, one of the pioneers of microsurgery in the United Kingdom, with whom he developed very close professional and personal relations. Mr. Bailey was appointed as a visiting faculty at GCRI, which he visited for 3 to 4 weeks every year, and during those visits he performed free flaps for cancer reconstruction, predominantly the radial artery and latissimus dorsi, apart from complex hand surgeries and head and neck reconstruction. This teamwork continued for approximately 8 years from 1976 to 1984. During this period, one of the earliest microsurgical training workshops was held at GCRI.

Dr. Jaju continued to work at GCRI till 2002, and toward the later part of this period I joined him at GCRI. Later, he continued private practice until coronavirus disease 2019 (COVID-19), in March 2020.

Dr. Jaju had been closely associated with the working of the executive committee of the APSI. He was the secretary, and then the President of APSI in 1987, when he organized the conference at twin locations of Ahmedabad – Mt. Abu ( Fig. 4 ). He worked with Dr. Goleria toward reframing the constitution of APSI in its current form. He was also closely associated with the APSI Trust, first as a member and then as executive Trustee, for many years to come.

**Fig. 4:**
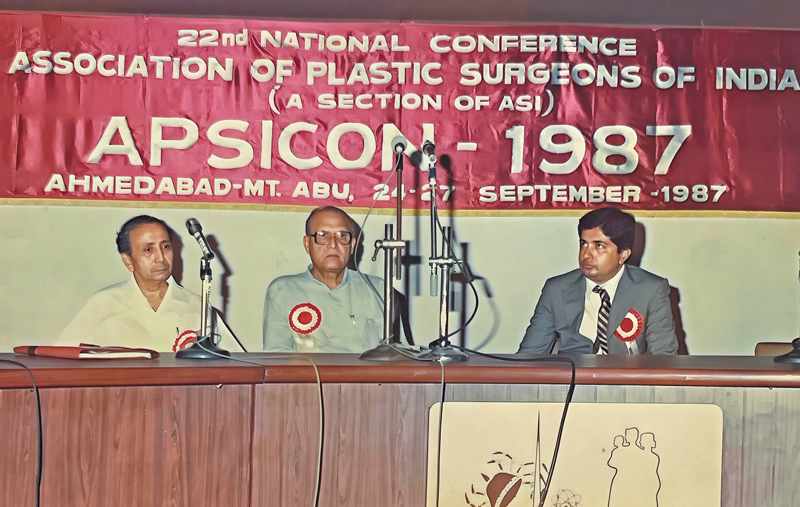
Dr. J.R. Jaju, Dr. Antia, and Dr. Vyas at APSICON 1987 (L to R).

He actively participated at the international and national conferences. At the APSI conferences he encouraged the junior colleagues for their active participation, and toward this, he donated funds at the APSI Chandigarh national conference in 1985, for initiating the “APSI Best Paper Award,” which is currently awarded at every APSI National Conference.

Dr. Jaju was very humble and easily approachable; for him patient care and well-being was supreme. His work spoke on his behalf. Once when Prof. Shrikant Lagvankar, who had worked with him as his junior, asked him for his visiting card, he smiled and said, “For me my face is my visiting card.” Plastic surgery in the state of Gujarat had become synonymous with the name Dr. J. R. Jaju.

He had done original surgical work in treatment of filarial lymphedema. He had special interests and did extensive work on congenital facial clefts, congenital constriction rings, high tension electrical injuries, and anorectal anomalies as well.

He was also associated with the Lymphology Association of India, the Indian Society of Surgery of the Hand, and the National Association of Burns India. He was actively involved with the local and state medical association bodies. He was involved in academic activities of the Indian Medical Association (IMA), at the state level, and played a major role in developing the education structure for the IMA College of General Practitioners, in rural and remote areas of Gujarat. He was the founder President of the Gujarat Association of Plastic Surgeons ( Fig. 5 ).

**Fig. 5:**
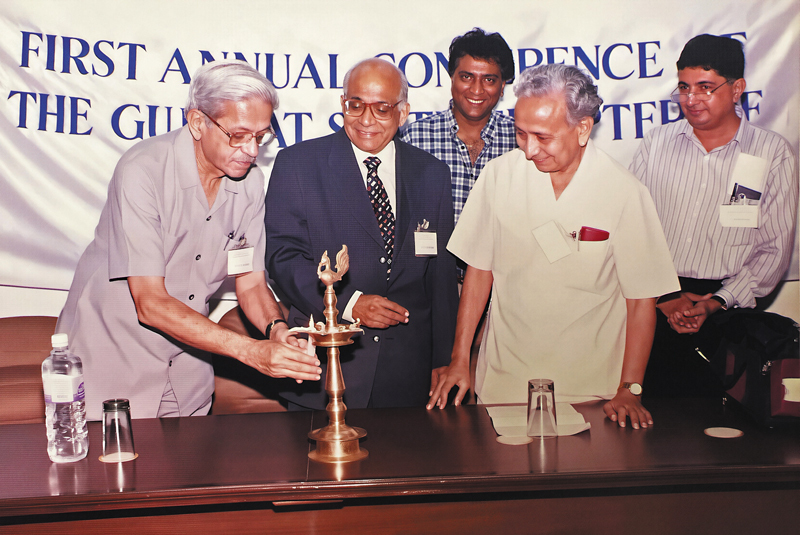
Dr. Goleria, Dr. Tambwekar, Dr. Nimish Patel, Dr. J.R. Jaju, and Dr. Vyas at the inaugural conference of Gujarat Plastic Surgery Association.

He contributed to charitable initiatives by actively organizing surgical camps and supporting patients through various Trusts. He strongly advocated that charity should be done through whatever *you* can do best. Since his early practicing years, he provided pro bono plastic surgery treatment to numerous underprivileged patients, through his actively operating Charitable Trust, which continues to date. He has extensively funded primary and secondary school education and its infrastructure development in his native village of Erandol, Maharashtra.

Outside of his professional life, Dr. Jaju was a person of deep curiosity and warmth, whose interests reached far beyond the boundaries of medicine. Friends, patients, and colleagues alike, speak of his honesty and unwavering sense of duty.

Through the many chapters of his life, Dr. Jaju balanced ambition with humility, and innovation with compassion. He paid close attention to details in every task that he undertook. He was a forthright person, who never faltered to call a spade a spade. He had a unique quality of “ *letting go* ” and moving forward, finding a path, even in the most adverse situation. For an adverse event he would say, “ *Past is past, take it as a bad dream, learn from it, and move on* .” He lived a simple, happy, and content life ( Fig. 6 ).

**Fig. 6:**
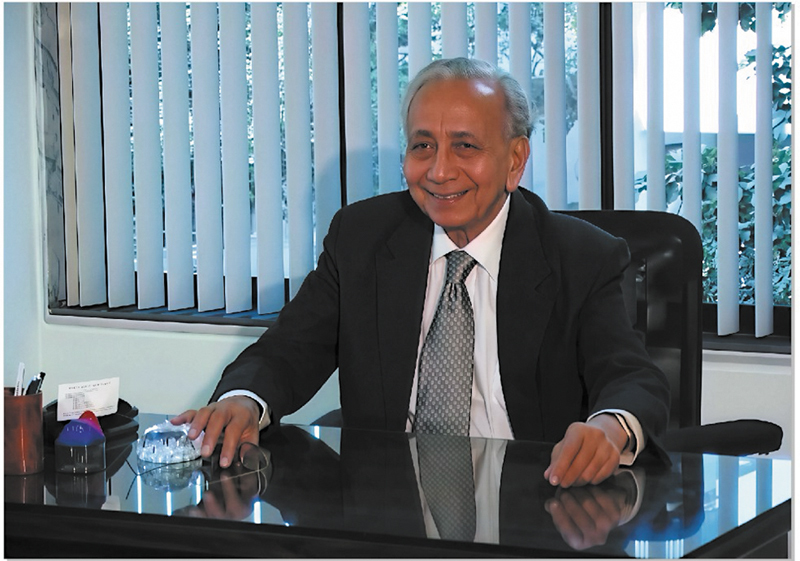
His warm smile always welcomed everyone who visited him.

He was very passionate about gardening and was greatly involved with plants. His day would not end without him having worked in the garden at home, for two hours every single day, taking personal care of the smallest details about each plant ( Fig. 7 ).

**Fig. 7:**
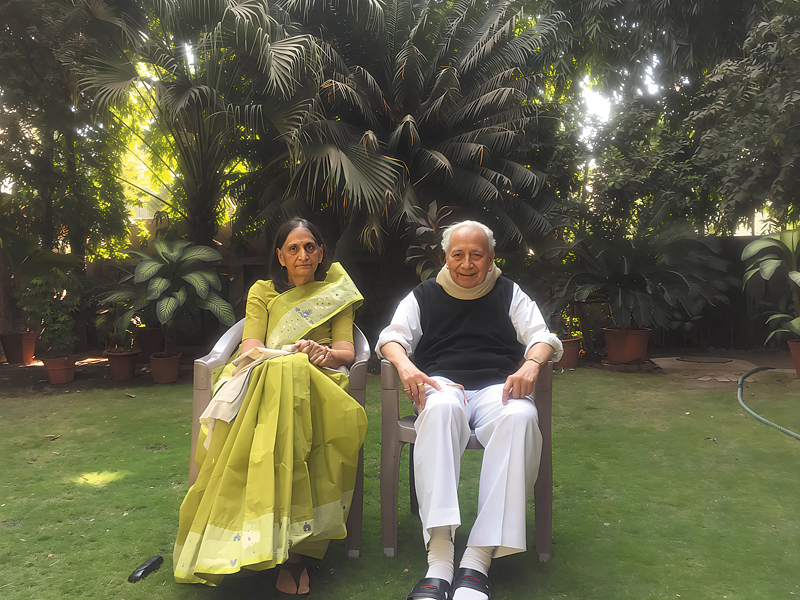
Dr. J. R. Jaju with wife Mrs. Chitra Jaju at home.

Till his wife, Chitra, died in 2019, he was actively involved in patient care and attended the operating theater every day. Since COVID-19 lockdown, at age of eighty-eight, he stopped his clinical work completely. Three days before he died, he told us that his time has come, and soon he shall leave. On the last day, September 8, 2023, he asked me to take him to his bed, held our hands, and warmly looked at us. I said, “Dad your time has come.” He closed his eyes for the last time, took a couple of breaths, and silently moved on. That was the most peaceful death, I have ever seen. A simple life, flowering into a gracious passing beyond. He is survived by his daughter, Dr. Rina Jaju and me.

His legacy lives on in the generations of medical professionals and people whose paths he illuminated through his example; but more importantly, his true legacy lies in the innumerable lives he has transformed—the burns survivor returning to school, the accident victim reclaiming his livelihood, the child smiling confidently after reconstructive surgery, and the cancer survivor living a better quality of life after reconstructive surgery.

## Tribute

Dr. Himanshu Vora, Consultant Plastic Surgeon, Ahmedabad

Dr. J. R. Jaju passed away on September 8, 2023. With his passing away plastic surgery fraternity has lost a legend not only in Gujarat but in India too. The word plastic surgery had become synonymous with his name in Gujarat, as he was the first plastic surgeon to start Plastic Surgery Department in Gujarat at Civil Hospital, Ahmedabad way back in 1964. Along with Dr. Puranik, the department scaled new heights, and all varieties of plastic surgeries were performed benefiting all strata of society. He established Reconstructive Surgery Unit in GCRI for post-cancer head and neck reconstruction in 1975.

I consider myself fortunate to have had the opportunity of working with him in his private hospital and at GCRI, Ahmedabad.

He had extremely strict work ethics and never allowed any compromise, be it surgery or simple dressing, and he would see it to its very completion.

He always kept himself updated with ongoing advances in the plastic surgery field by actively participating in national and international conferences as well as subscribing to the Plastic and Reconstructive Surgery (PRS) and British Journal of Plastic Surgery (BJPS) Journals.

Besides congenital anomalies, correction of post-burn deformities, extremity and faciomaxillary trauma, and burns management, the wide range of surgeries he executed were exstrophy of bladder, anorectal anomalies, correction of prognathism, nasofrontal encephalocele, and cosmetic surgeries. Excellent results of cleft lip and palate repair and hypospadias repair with Denis Brown techniques speak volumes of his skill.

The fact that he had special interest in microsurgery was evident when he had organized the workshop with Dr. Bruce Bailey at GCRI, Ahmedabad. He also devised a new procedure for lower limb lymphedema in the form of femoral artery ligation, which was recognized in McCarthy's Textbook of Plastic Surgery.

His contribution to the APSI as one of the early members is well known.

As a President of APSI he had organized National Conference at Ahmedabad and Mount Abu in 1987 and is remembered with fond memories.

One of my teachers, Late Prof. Udayan Vyas, and many from my generation, namely, Dr. Shrikant Lagvankar, Dr. Santosh Raibagkar, Dr. Hemant Saraiya, and me, were inspired by him in pursuing this specialty.

For me he had always been a father figure throughout my life, right from childhood to the present time. He had treated me as his son always, and in his loss, I lost my father for the second time. No words will be enough to express my gratitude to him and all his family members because it was due to their support and love that I have progressed to my present position.

I am incredibly happy to see his legacy continued by Hemen Jaju, his son.

Once again, I pay my sincere tributes to him.

